# Landing Error Scoring System: Data from Youth Volleyball Players

**DOI:** 10.1016/j.dib.2022.107916

**Published:** 2022-02-03

**Authors:** Weerawat Limroongreungrat, Christopher Mawhinney, Suthasinee Kongthongsung, Chatchadaporn Pitaksathienkul

**Affiliations:** a College of Sports Science and Technology, Mahidol University, Thailand; b Sports Science Bureau, Department of Physical Education, Ministry of Tourism and Sports Thailand

**Keywords:** Youth volleyball, Landing error scoring system, Screening tool, Screening test, Sport injuries

## Abstract

The Landing Error Scoring system (LESS) is a reliable screening tool for Anterior Cruciate Ligament (ACL) injury. The test is focused on biomechanical errors of landing motion and is used to evaluate the risk of knee injuries in several sports, such as football and basketball, which involve repeated jumping demands. Presently, available LESS data from youth volleyball players is limited, thus screening for injury risk has not been comprehensively undertaken in this cohort. The LESS is typically performed by jumping from a box while video motion in 2 sagittal and frontal planes is recorded, with the jump landing rated against 17-items. A total of 233 players performed three jump landing trials resulting in a total of 1398 videos being recorded. Each LESS score item was rated by two physical therapists and one sports scientist and the data were separated into four separate LESS score categories: excellent (≤4), good (4 - 5), moderate (5 - 6), and poor (>6). Descriptive analysis (percentage) was employed to describe the data, with scores subdivided by gender. The data may be applied to identify youth volleyball players at potential risk of sustaining a lower body injury from poor landing biomechanics.

## Specifications Table

**Table:** 

Subject	Sport Sciences, Therapy and Medicine
Specific subject area	Landing Error Scoring System (LESS) Youth Sports Screening test
Type of data	Table Chart
How data were acquired	Two digital cameras (Sony HDR-P675 & Canon HF-M41) were used to record jump-landing motion. A VLC media player was used for video analysis.
Data format	Raw
Parameters for data collection	[Provide a brief description of which conditions were considered for data collection. Max 400 characters] Youth volleyball players in the same age group from different schools were screened via LESS test. Two cameras were used to record motion in the front and side views.
Description of data collection	[Provide a brief description of how these data were collected. Max 600 characters] Youth volleyball players performed three jumps from a 30 cm box height, jumping forward at least 50% of their height. Once the feet contacted the ground, the players were required to immediately jump upwards as high as possible. The motion in the sagittal and frontal planes were recorded. The trials of the LESS score were rated by three raters, with the scores averaged (over 3 trials) and analyzed.
Data source location	See Table 2
Data accessibility	Repository name: LESS Scores Youth Volleyball Data identification number: 10.17632/vff594767s.1 Direct URL to data: https://data.mendeley.com/datasets/vff594767s/draft?a=543beb7f-17f0-4caa-9a90-8bf5825cf363

## Value of the Data

• These data are useful for screening biomechanical risk factors of anterior cruciate ligament (ACL) injuries in youth volleyball players [1], [2], [3]
• The data may benefit physiotherapists, sports scientist and coaches when applying the LESS screening to test athletes; helping to evaluate ACL injury risk.
• The data provide details of each LESS item, which may useful as a reference to identify the item/s which incur the most error (when landing) during the test in this cohort. Therefore, correcting and training to reduce these errors may reduce potential injury occurrence.

## 1 Data Description

The Data reported in this article was collected from Landing Error Scoring System (LESS) screening tests, which were performed by youth volleyball players from schools located across the central regions of Thailand. A repository dataset of 233 players was composed from the 17-item LESS scores and includes: knee flexion angle at initial contact (L1), hip flexion angle at initial contact (L2), trunk flexion at initial contact (L3), ankle plantar flexion at initial contact (L4), knee valgus at initial contact (L5), lateral trunk flexion (L6), stance width – wide (L7), stance width – narrow (L8), foot position – toe In (L9), foot position – toe out (L10), symmetric initial foot contact (L11), knee flexion displacement (L12), hip flexion at max knee flexion (L13), trunk flexion at max knee flexion (L14), knee valgus displacement (L15), joint displacement (L16) and overall impression (L17) [1].

Descriptive data of participant characteristics are presented in Table 1.

**Table 1:** Participant characteristics (Mean ±SD).

	*n*	Age (years)	Weight (Kg)	Height (m)	BMI (Kg/m^2^)
Male	97	12.21 ± 0.89	41.70 ± 12.93	1.48 ± 0.11	18.75 ± 4.16
Female	136	11.89 ± 0.99	41.8 ± 11.27	1.49 ± 0.09	18.64 ± 3.68
Total	233	12.02 ± 0.96	71.76 ± 11.97	1.48 ± 0.10	18.69 ± 3.88

**Fig. 1:**
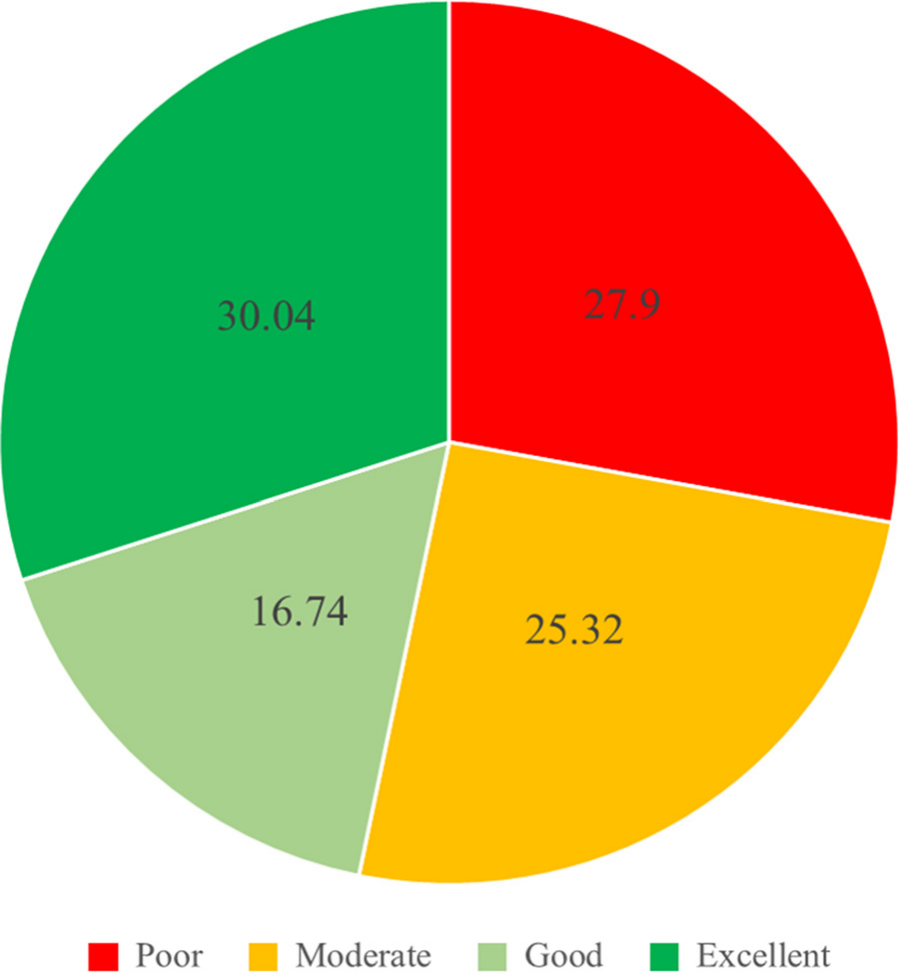
The overall percentage LESS score data in each category.

**Fig. 2:**
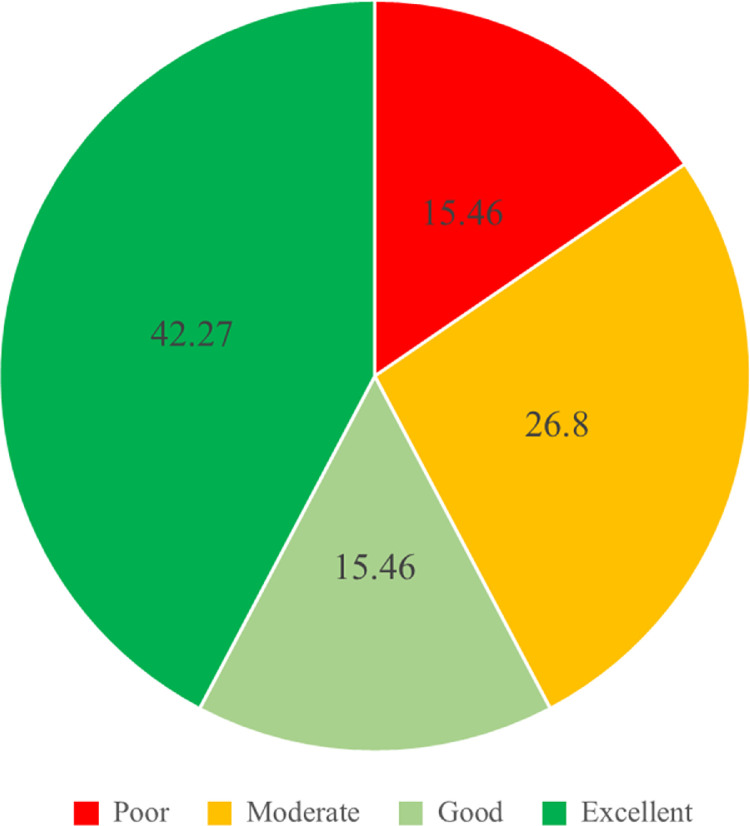
The percentage LESS score data in each category in the male group.

**Fig. 3:**
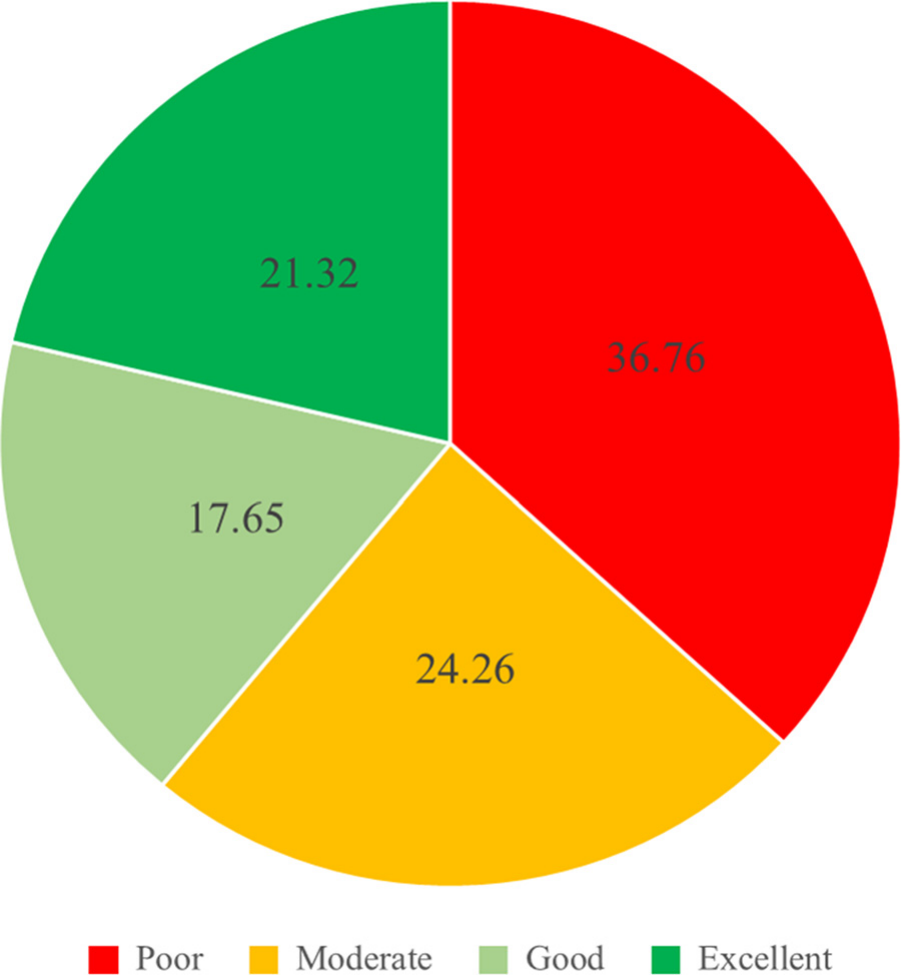
The percentage LESS score data in each category in the female group.

The LESS scores were divided into 4 categories; excellent (≤4), good (>4 to ≤5), moderate (>5 to ≤6), and poor (>6). Figure 1, 2 and 3 report the LESS scores based upon the 4 categories, with scores subdivided by overall total and gender.

## 2 Experimental Design, Materials and Methods

### 2.1 Participants

The participants were youth volleyball players who competed in junior school volleyball competitions and from central region schools in Thailand (see Table 2). Participants were included if they were aged 10–13 years old, male or female, and had been training for volleyball competition for at least 2 years. Participants were excluded if they had sustained a musculoskeletal injury within the previous 3 months prior to commencing experimental testing or encountered any other injury that could obstruct their performance at the time of testing. The participant characteristics of the 233 participants (male = 97, female = 136) are shown in Table 1. The purpose and procedures of the study were explained to the participants and responsible guardian. All participants and guardians read and signed informed consent forms that were approved by the Mahidol University Central Institutional Review Board (COA no.2016/118.1209).

**Table 2:** Source location of collected data.

Institution	Country	Location
Ban Band Kung School	Thailand	City/Town/Region: Supanburi Latitude and longitude: 14° 27′ 18.5754″, 100° 0′ 29.1414″
Wat Thepitak School	Thailand	City/Town/Region: Supanburi Latitude and longitude: 14° 8′ 28.287″, 99° 56′ 58.3764″
Ban Sap Sanun School	Thailand	City/Town/Region: Supanburi Latitude and longitude: 14° 53′ 0.042″, 101° 16′ 55.6788″
Ban Plangwai-KhunKlung School	Thailand	City/Town/Region: Chachoengsao Latitude and longitude: 13° 31′ 16.5102″, 101° 27′ 48.5166″
Wat Pa Community School	Thailand	City/Town/Region: Nakhon Nayok Latitude and longitude: 14° 17′ 12.5478″, 101° 4′ 7.446″
Ban Khao Hua Na School	Thailand	City/Town/Region: Nakhon Nayok Latitude and longitude: 14° 20′ 36.819″, 101° 6′ 44.1858″
Wat Bot Karong School	Thailand	City/Town/Region: Nakhon Nayok Latitude and longitude: 14° 10′ 33.8406″, 101° 9′ 4.0638″
Wat Lek Thammakit School	Thailand	City/Town/Region: Nakhon Nayok Latitude and longitude: 14° 10′ 51.1782″, 101° 4′ 6.33″
Bung Khao Yon School	Thailand	City/Town/Region: Pathum Thani Latitude and longitude: 14° 4′ 55.4262″, 100° 41′ 15.828″
Wat Don Thong School	Thailand	City/Town/Region: Chachoengsao Latitude and longitude: 13° 40′ 5.4402″, 101° 5′ 54.8514″
Talat Bang Bo School	Thailand	City/Town/Region: Chachoengsao Latitude and longitude: 13° 36′ 17.8272″, 101° 14′ 54.8802″
Ban Khlong Song School	Thailand	City/Town/Region: Chachoengsao Latitude and longitude: 13° 33′ 36.7374″, 101° 21′ 13.9248″
Wat Nong Wa School	Thailand	City/Town/Region: Saraburi Latitude and longitude: 14° 39′ 54.3666″, 100° 53′ 27.9774″
Ban Nong Pla Lai School	Thailand	City/Town/Region: Kanchanaburi Latitude and longitude: 14° 38′ 1.734″, 99° 32′ 9.6894″
Wat Nong Khu School	Thailand	City/Town/Region: Lopburi Latitude and longitude: 14° 56′ 46.014″, 100° 37′ 36.5196″
Chareondee Wittaya School	Thailand	City/Town/Region: Pathum Thani Latitude and longitude: 13° 57′ 20.181″, 100° 46′ 26.8674″
Wat Thung Din Kho School	Thailand	City/Town/Region: Saraburi Latitude and longitude 14° 22′ 12.6834″, 100° 49′ 44.7744″
Ban Nong Kan Cham School	Thailand	City/Town/Region: Nakhon Nayok Latitude and longitude: 14° 25′ 34.3″, 101° 00′ 06.0″

### 2.2 Procedure

Two video cameras were positioned perpendicular to the plane of motion (sagittal and frontal planes) and set at 3.64 m from the area of landing [1]. Prior to commencing the test, masking tape was placed at 50% of the player's height on the floor in front of a box that was 30 cm in height. After a warm-up, participants stood atop of the box and jumped forward beyond the tape marker, immediately jumping upwards as high as possible upon foot contact with the ground. A total of three jump landing motions were recorded with a 2 min rest period between each trial. A total of 1398 videos were recorded using a VLC media player to allow the evaluation of LESS scores by 3 raters (2 physical therapists and a sports scientist). The LESS has 2 different versions, a fulI version (17 items) and a real-time version (10 items aka LESS-RT), with both versions possessing high reliability [4,5]. In this report, the full version was employed. Microsoft Excel (Microsoft corp., Redmond, WA) was used to estimate ICC and 95% confident intervals based on mean-ratings (*k* = 3), absolute-agreement, and a 2-way mixed-effects model. The ICC indicated high interrater reliability (ICC = 0.81, 95% CI 0.708–0.883) [2].

### 2.3 Statistical analysis

The LESS scores obtained from each participant's three trials were averaged. Descriptive statistical analysis was performed and presented using google data studio (Google Inc., Mountainview CA) (https://datastudio.google.com/s/pIUH9cEmAgM).

## Ethics Statement

All participants and guardians had the experimental procedures and associated risk and benefits fully explained prior to providing their informed consent to participate. All procedures were granted ethical approval from the Mahidol University Central Institutional Review Board (COA no.2016/118.1209).

## CRediT authorship contribution statement

**Weerawat Limroongreungrat:** Conceptualization, Methodology, Writing – review & editing. **Christopher Mawhinney:** Writing – review & editing. **Suthasinee Kongthongsung:** Data curation. **Chatchadaporn Pitaksathienkul:** Data curation.

## Declaration of Competing Interest

The authors declare that they have no known competing financial interests or personal relationships which have, or could be, perceived to have influenced the work reported in this article.
